# Discrete-Event Simulation to Model the Thrombolysis Process for Acute Ischemic Stroke Patients at Urban and Rural Hospitals

**DOI:** 10.3389/fneur.2021.746404

**Published:** 2021-10-29

**Authors:** Tessa Bulmer, David Volders, John Blake, Noreen Kamal

**Affiliations:** ^1^Department of Industrial Engineering, Faculty of Engineering, Dalhousie University, Halifax, NS, Canada; ^2^Interventional and Diagnostic Neuroradiology, QEII Health Sciences Centre, Nova Scotia Health, Halifax, NS, Canada; ^3^Department of Radiology, Faculty of Medicine, Dalhousie University, Halifax, NS, Canada

**Keywords:** acute ischemic stroke (AIS), door-to-needle time (DNT), tissue plasminogen activator (tPA), thrombolysis, stroke pathways, discrete-event simulation (DES), urban, rural

## Abstract

**Background:** Effective treatment with tissue plasminogen activator (tPA) critically relies on rapid treatment. Door-to-needle time (DNT) is a key measure of hospital efficiency linked to patient outcomes. Numerous changes can reduce DNT, but they are difficult to trial and implement. Discrete-event simulation (DES) provides a way to model and determine the impact of process improvements.

**Methods:** A conceptual framework was developed to illustrate the thrombolysis process; allowing for treatment processes to be replicated using a DES model developed in ARENA. Activity time duration distributions from three sites (one urban and two rural) were used. Five scenarios, three process changes, and two reductions in activity durations, were simulated and tested. Scenarios were tested individually and in combinations. The primary outcome measure is median DNT. The study goal is to determine the largest improvement in DNT at each site.

**Results:** Administration of tPA in the imaging area resulted in the largest median DNT reduction for Site 1 and Site 2 for individual test scenarios (12.6%, 95% CI 12.4–12.8%, and 8.2%, 95% CI 7.5–9.0%, respectively). Ensuring that patients arriving via emergency medical services (EMS) remain on the EMS stretcher to imaging resulted in the largest median DNT improvement for Site 3 (9.2%, 95% CI 7.9–10.5%). Reducing both the treatment decision time and tPA preparation time by 35% resulted in a 11.0% (95% CI 10.0–12.0%) maximum reduction in median DNT. The lowest median and 90^th^ percentile DNTs were achieved by combining all test scenarios, with a maximum reduction of 26.7% (95% CI 24.5–28.9%) and 17.1% (95% CI 12.5–21.7%), respectively.

**Conclusions:** The detailed conceptual framework clarifies the intra-hospital logistics of the thrombolysis process. The most significant median DNT improvement at rural hospitals resulted from ensuring patients arriving via EMS remain on the EMS stretcher to imaging, while urban sites benefit more from administering tPA in the imaging area. Reducing the durations of activities on the critical path will provide further DNT improvements. Significant DNT improvements are achievable in urban and rural settings by combining process changes with reducing activity durations.

## Introduction

Stroke is a devastating disease, but is treatable with alteplase or tissue plasminogen activator (tPA) (1) and endovascular thrombectomy (EVT) (2–6). Tissue plasminogen activator has been a proven treatment for acute ischemic stroke (AIS) since 1995 (1), and is widely available in urban and rural hospitals. A person having a stroke loses approximately 1.9 million neurons every minute (7), leading to the popular motto “time is brain.” Acute ischemic stroke patients should be treated with tPA as rapidly as possible for maximal benefit (8, 9), as the effectiveness is highly time dependent (10). Door-to-needle time (DNT) is a critical measure of hospital efficiency linked to patient outcomes and is defined as the time from a patient's hospital arrival to the start of tPA treatment. Fast treatment with tPA has been reported in many urban hospitals, but rural hospitals struggle to reduce treatment times (11, 12). A recent study analyzing the thrombolysis process for AIS in urban and rural hospitals highlighted that physician comfort, resource availability, and frequency of treating AIS patients were factors that lead to an inequality in treating patients quickly in rural settings (13). There are key resource differences between urban and rural centers. For instance, rural sites may only have computed tomography (CT) technologists on-call during out of hours, and emergency department (ED) physicians are making the treatment decision, instead of neurologists (13).

Several process changes can reduce DNT (12, 14), but these changes are difficult to trial and implement and could disturb established care pathways. Discrete-event simulation (DES) provides a safe and efficient way to model processes and determine the impact of process changes. Operations research techniques, such as simulation, have become a common analytical problem-solving method used in healthcare. Simulation has been amply used to address challenges in ED patient flow optimization (15). Further, simulation allows complex healthcare systems with stochastic elements, such as stroke pathways, to be replicated to provide insights and recommendations for improvements. Discrete-event simulation has been shown to be an effective approach applied to pre-hospital (16, 17), intra-hospital (18–21), and both pre and intra-hospital aspects of the acute stroke pathways (22–29). Discrete-event simulation literature also spans to areas such as operation of a stroke unit (30), the impact of additional comprehensive stroke centers for EVT (31), and AIS patient disability post-hospital (32). Outcome measures among DES studies include: resource optimization (21), thrombolysis eligibility (16), utilization rate of thrombolysis or intra-arterial thrombectomy (22, 23, 27, 28), and patient outcomes (22, 23, 28, 32), amongst others. Improving thrombolysis rates and patient outcomes were the most common aims of the DES studies for acute stroke care found in the literature.

Several studies have illustrated the key activities involved in either the pre-hospital or intra-hospital aspects of acute stroke care (16, 23, 24, 27–29, 33). These studies include overviews of intra-hospital thrombolysis steps and also identify different pathway types but lack detail regarding intra-hospital activities and sequences. Additionally, process differences based on pathway type or out of hour resource differences are not well-defined in the literature. We address these gaps by developing a conceptual framework of the intra-hospital aspect of the thrombolysis treatment process by analyzing three urban and rural sites. The framework defines intra-hospital treatment processes, based on patient pathway type, shows resource availability differences in out of hour operations, provides further detail of process activities and sequences, and highlights when potential delays may be encountered. It is important to fill these gaps to provide clarity of intra-hospital logistics, which can lead to solutions to reduce DNT. The study objectives are: 1) to provide a detailed conceptual framework of the thrombolysis process, focusing on intra-hospital activities; and 2) to assess the potential impact of process improvements that can result in faster DNTs when applied to urban and rural settings using a DES model.

## Methods

A conceptual framework was developed first to provide a generalized model for tasks involved in the thrombolysis process within both urban and rural hospitals. This conceptual model was then used to develop a DES model that can be used for both urban and rural hospitals, based on various inputs. This paper includes descriptions of the conceptual framework, the DES model, as well as the test scenarios applied to the three included sites. The authors declare that all supporting data are available within the article and its Supplementary Material.

### Development of Conceptual Framework

A qualitative study was conducted in Nova Scotia, Canada, to understand the thrombolysis treatment process in urban and rural hospitals. This study provided the foundation for the current research; the full details of this study are published elsewhere (13). There was one urban and two rural sites chosen to enable comparisons between urban and rural hospitals. The respective site distinctions, local target median DNTs, and current median DNTs are detailed in the published qualitative study in the *Site Context* section (13). The results from that study provided the necessary pathway specifics to create a detailed process map for each site (13), and estimations of activity durations. These process maps were used in this study to develop a conceptual framework of the thrombolysis process in urban and rural hospitals and the DES.

**Figure 1:**
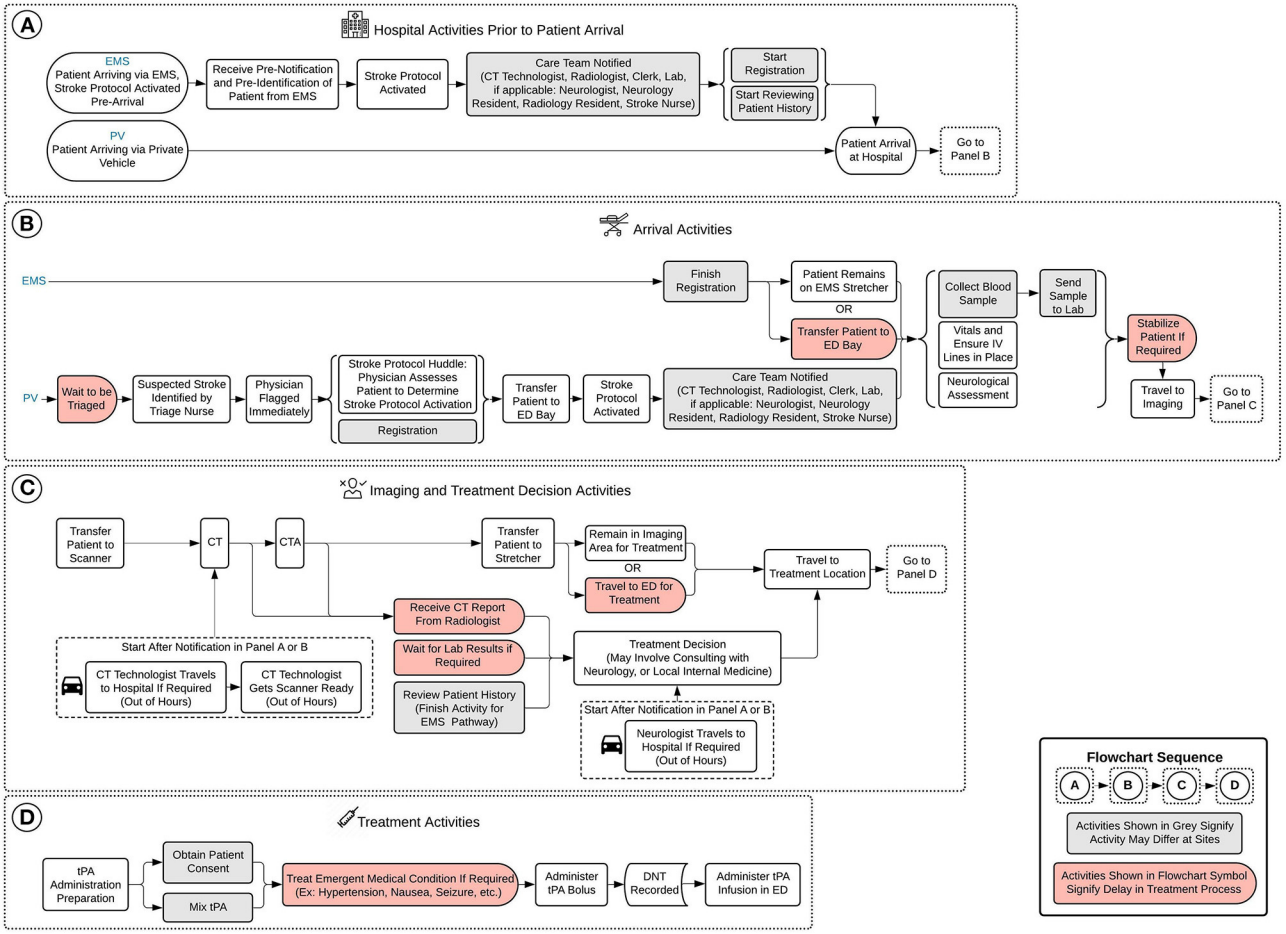
Conceptual framework of model. **(A)** Hospital activities prior to patient arrival, **(B)** arrival activities, **(C)** imaging and treatment decision activities, and **(D)** treatment activities. EMS, emergency medical services; PV, private vehicle; CT, computed tomography; ED, emergency department; IV, intravenous; CTA, computed tomography angiography; tPA, tissue plasminogen activator; DNT, door-to-needle time.

The conceptual framework shows the intra-hospital aspects of the thrombolysis process divided into four panels as shown in Figure 1: (Figure 1A) hospital activities prior to patient arrival; (Figure 1B) arrival activities; (Figure 1C) imaging and treatment decision activities; and (Figure 1D) treatment activities. Figure 1A illustrates the intra-hospital activities that take place during the pre-hospital stage, while the remaining panels represent the hospital-based stage of the treatment process. The framework considers two patient treatment pathways: patients arriving via emergency medical services (EMS), where the stroke protocol has been activated at the hospital prior to the patient's arrival, and patients arriving via private vehicle (PV). Figures 1A,B display activities in terms of treatment pathways, as the logistics for this portion of the process are pathway dependent. Figure 1A illustrates efficiencies of the EMS treatment pathway, as several activities can begin in-hospital prior to the patient's arrival. Figure 1B shows the evident difference in activities required to be completed upon arrival between the EMS and PV pathways. When patients enter Figure 1C of the treatment process, imaging and treatment decision activities, the process is identical for both patients arriving via EMS and PV. Activities shown in gray in Figure 1 signify the activity may differ by site regarding how and when the activity is executed, as the healthcare professionals responsible for completing the activities may differ. Activities shown in red in Figure 1 signify a potential delay in the treatment process. While patient-related delays cannot be entirely avoided, they can be minimized with appropriate anticipation and preparation. However, system delays (i.e., delays not specifically related to the patient) can, and should, be eliminated to achieve more streamlined treatment.

### Simulation Model

A DES model was developed based on the conceptual framework shown in Figure 1 and the previously developed process maps (13), and applied to the three sites to verify the model and provide context for the test scenarios. The model provides representation of the intra-hospital aspect of the thrombolysis process for urban and rural hospitals. The model includes the two acute stroke pathways noted in Figure 1. The strengthening the reporting of empirical simulation studies guidelines developed for discrete-event simulations (STRESS-DES) was consulted to report key aspects of the DES model in the following domains: objectives, logic, data, experimentation, implementation, and code access (34).

#### Objectives

The DES aims to replicate current practice of the thrombolysis treatment process of AIS patients for each site and gives the ability to assess the potential impact of process improvements that can result in reduced DNT.

##### Test Scenarios

In addition to current baseline treatment processes, five scenarios were tested for each site. The scenarios are three process changes and two reductions in activity durations. Process change scenarios entail implementing a change in a site's current thrombolysis treatment process, while reduction in activity duration scenarios reduces the amount of time taken to execute a specified activity. The five scenarios are defined in Table 1, along with defining current site processes. The process changes were chosen because they are well-supported in previous studies to have reduced DNTs. Activities chosen to test the effect of a reduction in duration were selected where there is considerable variability in activity length of time, and evidence that times can be reduced with standardized protocols. The scenarios were tested individually, and in combinations.

**Table 1:** Test scenarios and current site processes.

**Test scenarios**	**Is the test scenario already implemented at the site? (i.e., included in site baseline)**		
	***Site 1 (Urban)***	***Site 2 (Rural)***	***Site 3 (Rural)***
(P1) Patients arriving via EMS remain on EMS stretcher to imaging	Yes	Yes	No (patient transferred to ED bay)
(P2) Administration of tPA in imaging area (regular and out of hours)	No (during regular hours only)	No (administered in ED)	No (administered in ED)
(P3) Pre-registration of patients arriving via EMS	Yes	Yes	No (completed upon arrival)
(R1) Reduce treatment decision time by 35%	–	–	–
(R2) Reduce tPA administration preparation time by 35%	–	–	–

*EMS, emergency medical services, tPA, tissue plasminogen activator. “Yes” indicates the scenario is already implemented at the site, while “No” indicates the scenario is not a current site process and the current site process is subsequently defined*.

The following lists and details the five test scenarios that were run and applied to the three included sites in this study:

*Process change 1 (P1): Patients arriving via EMS remain on EMS stretcher to imaging* (12, 14, 35–39)—This scenario models the impact of keeping a patient arriving via EMS on the EMS stretcher, as opposed to transferring the patient to an ED bay to travel to imaging.

*Process change 2 (P2): Administration of tPA in imaging area (regular and out of hours)* (14, 35, 36, 40–42)—This scenario models the administration of the tPA bolus in the imaging area, as opposed to administration in the ED following imaging. This scenario eliminates the travel time from imaging to the ED prior to the administration of the bolus and has a reduced tPA administration preparation activity time.

*Process change 3 (P3): Pre-registration of patients arriving via EMS* (14, 40, 41)—This scenario models the pre-registration of patients traveling via the EMS pathways, as opposed to starting the registration process when the patient arrives at the hospital.

*Reduction in activity duration 1 (R1): Reduce treatment decision time by 35%—*This scenario models a reduction in the time taken for the physician to decide whether the patient will receive thrombolysis treatment by 35%.

*Reduction in activity duration 2 (R2): Reduce tPA administration preparation time by 35%—*This scenario models a reduction in the time taken to complete the preparation required before administering tPA by 35%. Tissue plasminogen activator administration preparation encompasses activities such as: obtaining the drug, estimating the patient's weight, calculating the correct dosing, mixing the tPA, and programming the tPA administration pump.

##### Outcome Measures

The primary outcome measure for this study was median DNT for patients treated with thrombolysis, and the measure of variance for this outcome was interquartile range (IQR). The reduction in IQR was also reported. A critical outcomes measure that was included was 90^th^ percentile DNT for patients treated with thrombolysis. This measure is important as it shows the DNT where most (90% of patients) are treated within, and there has been previous justification for the use of this measure in acute stroke processes (43). The current Canadian Guidelines state that the 90^th^ percentile for DNT should be 60 min (44).

##### Statistical Analysis

The Mann-Whitney U-test and the Chi-Square Goodness-of-Fit test were performed for continuous and categorical variables, respectively. Minitab Statistical Software for Windows (Minitab, State College, PA, version 19) was used for all statistical analysis. A *p*-value of < 0.05 was considered statistically significant.

#### Logic

The model considers variation in patient pathway and activity durations using probability distributions and employs a stochastic patient arrival rate schedule. Once patients arrive, the model distributes patient pathways with approximately 80% of patients arriving via EMS, and 20% arriving via PV. Activity duration distributions are based on estimations from healthcare professionals at the included sites. A time study would be required to verify the activity duration distributions. The modeled process baseline median DNTs matched the sites' current median DNTs, which provided some verification of the estimates. The arrival rate schedule was approximated from Site 1 data to more accurately replicate hourly volumes in which AIS patients arrive at a hospital. As aggregate data showed that Site 2 and Site 3 had lower ischemic stroke volumes compared to Site 1 (with Site 3 encountering the least amount of AIS patients), their arrival rates were adjusted accordingly. The time and day of a patient's arrival in the system can impact their treatment pathway due to resource availability. Regular hours are defined as 8:00 a.m. to 4:00 p.m., Mondays to Fridays, while out of hours are considered all times outside of regular hours. The schedule generates approximately 35% of arrivals in regular hours and 65% during out of hours as determined from our input data. The schedule specifies that the majority of arrivals occur between 8:00 a.m. and 8:00 p.m., with few arrivals between 12:00 a.m. and 8:00 a.m. Human resource and the resulting treatment process differences between regular and out of hours are defined for each site in the *Section 2 Treatment Process Results* section of the qualitative study (13). In addition to the conceptual framework shown in Figure 1, ARENA simulation images (Supplementary Figures 1–6) and model activity duration distributions for process baselines and all test scenarios (Supplementary Table 1) can be found in Supplementary Material for further clarity.

#### Data

The model activity duration distributions (Supplementary Table 1) and arrival rate schedules (Supplementary Table 2) are detailed in Supplementary Material. Additionally, verification techniques (Supplementary Tables 3–5), sensitivity analysis results (Supplementary Table 6), and number of replications analysis results (Supplementary Table 7) are detailed in Supplementary Material. Arrival rate and pathway type verification results are shown in Table 2.

**Table 2:** Site 1 model verification of arrival rate and pathway types.

**Time period**	**Desired rate**	**Model rate**	**Desired proportion**	**Desired volume**	**Model volume**	***p*-Value**
12:00 a.m.−2:00 a.m.	0.00428	0.01308	0.0310	93.7	79	0.169
2:00 a.m.−4:00 a.m.	0.00321	0.01291	0.0233	70.4	78	
4:00 a.m.−6:00 a.m.	0.00321	0.01274	0.0233	70.4	77	
6:00 a.m.−8:00 a.m.	0.00642	0.02416	0.0465	140.5	146	
8:00 a.m.−10:00 a.m.	0.01391	0.05660	0.1008	304.5	342	
10:00 a.m.−12:00 p.m.	0.01819	0.06207	0.1318	398.2	375	
12:00 p.m.−2:00 p.m.	0.01926	0.06869	0.1395	421.4	415	
2:00 p.m.−4:00 p.m.	0.01712	0.05776	0.1240	374.6	349	
4:00 p.m.−6:00 p.m.	0.01712	0.06223	0.1240	374.6	376	
6:00 p.m.−8:00 p.m.	0.01605	0.05925	0.1163	351.3	358	
8:00 p.m.−10:00 p.m.	0.01070	0.03608	0.0775	234.1	218	
10:00 p.m.−12:00 a.m.	0.00856	0.03443	0.0620	187.3	208	
**Definition of time period**	**Desired percentage of arrivals (%)**			**Model output (%)**		
Regular hours (Monday–Friday, 8:00 a.m.−4:00 p.m.)	35.4			34.5		
Out of hours (Monday–Friday, 4:00 p.m.−8:00 a.m., Saturday and Sunday)	64.6			65.5		
**Pathway type**	**Desired pathway distribution (%)**			**Model output (%)**		
EMS	80.0			79.5		
PV	20.0			20.5		

*EMS, emergency medical services; PV, private vehicle. Analysis was performed using the chi-square goodness-of-fit test*.

The DES has several assumptions and simplifications:

1. Due to the top prioritization of acute stroke patients within a hospital, it is assumed these patients will receive priority for the required resources upon arrival, and that the resources are always available.
2. Patient pathway type assignments (EMS or PV) are independent.
3. The EMS pathway always provides the hospital with pre-notification and pre-identification.
4. Activity durations are independent of each other.
5. All patients in the DES model are eligible to receive thrombolysis treatment.
6. The system may have only a single stroke protocol activation at a time.
7. The model assumes 100% stroke protocol compliance and does not consider personnel dependent variation. Although, it should be noted that waiting for a patient's lab results is not always required to determine the thrombolysis treatment decision, which is reflected in the duration distribution for this activity.

There are various reasons based on literature for the established DES assumptions that allow the model to produce meaningful results. Assumption 1 was established due to the Canadian triage and acuity scale (CTAS) level assigned to AIS patients in the qualitative study which provided the foundation for this work (13). Assumptions 2 and 4 are in agreement with a common DES model assumption made regarding acute stroke systems, which is independence of patient attributes (33). Assumptions 3 and 6 are considered appropriate based on expert-opinion from interview participants in the founding qualitative study (13) that indicated pre-identification and pre-notification are generally received for patients traveling via the EMS pathway, and that it would be a rare occurrence to have more than one stroke protocol activation simultaneously at a hospital. As this study focuses on the impact of process improvements on DNT, assumption 5 ensured all patients included in the model received tPA thus outputting a DNT. The final assumption 7 was established due to a lack of data regarding compliance variation, as well as allowing the maximum potential of a process improvement to be illustrated.

The following are model simplifications:

1. Activity durations are independent of the onset to arrival time, meaning durations do not change if a patient is approaching the 4.5-h window.
2. Only AIS patients are included; stroke mimics, and hemorrhagic stroke patients are excluded.
3. Ineligible thrombolysis candidates are not considered.
4. Stroke severity, patient age, and patient sex are not considered.

Certain variation in activities may be due to factors included in the assumptions and simplifications listed above; for example, milder stroke patients often have longer treatment times, due to the time taken to determine a diagnosis, and treatment decision. These variations in the associated activities are considered in the distribution of each relevant activity in the DES.

#### Experimentation

The model did not include a warm-up period due to the transient nature of the treatment process being modeled. As randomness exists in all simulation results, a number of replications analysis was conducted to determine the number of runs required to achieve a particular precision of the outcome estimate, summarized in Supplementary Table 7 in Supplementary Material. It was determined that model runs would consist of 30 replications. As real site median DTN data from Nova Scotia Health was provided for a period of 1 year, a replication length of 1 year was chosen for the model.

#### Implementation

The simulation model was developed using ARENA software (Rockwell Automation, Milwaukee, WI, version 16.00.00003).

#### Code Access

To obtain access to the developed ARENA model, please contact the corresponding author.

### Ethics

Ethics approval was obtained from the Nova Scotia Health Research Ethics Board (REB) for this study, with the REB file number 1025975.

## Results

The model was used to determine the impact of process changes and reduction in activity durations on DNT at each site in comparison to the site-specific baseline DNT. The actual median DNT for Site 1, Site 2, and Site 3 are 50.0, 40.0 and 77.5 min (June 2019–May 2020), with modeled baseline median DNT of 50.0 (IQR 45.4–53.8), 40.1 (IQR 38.7–48.0), and 74.0 (IQR 70.8–82.6) min, respectively. The results of the model baselines and test scenarios are summarized in Table 3, with the median DNT and IQR for the total 30 replications calculated for each scenario.

**Table 3:** Median DNT results by scenario for Site 1, Site 2, and Site 3.

	**Site 1 (Urban)**			**Site 2 (Rural)**			**Site 3 (Rural)**		
	***n***	**Median DNT (IQR) (min)**	***p*-Value**	***n***	**Median DNT (IQR) (min)**	***p*-Value**	***n***	**Median DNT (IQR) (min)**	***p*-Value**
**Process baseline**	3,021	50.0 (45.4–53.8)	–	780	40.1 (38.7–48.0)	–	405	74.0 (70.8–82.6)	–
**Process changes**									
P1	3,021	Current baseline	–	780	Current baseline	–	405	66.4 (63.6–75.7)	<0.0001
P2	2,984	43.7 (42.2–46.3)	<0.0001	856	36.6 (34.9–44.6)	<0.0001	405	71.0 (66.0–80.3)	<0.0001
P3	3,021	Current baseline	–	780	Current baseline	–	405	71.9 (68.6–80.9)	<0.0001
**Reduction in activity durations**									
R1	3,021	50.0 (45.4–53.8)	1.000	780	39.8 (38.5–48.0)	0.1430	405	72.3 (70.0–76.0)	<0.0001
R2	3,022	45.7 (42.0–49.1)	<0.0001	780	37.8 (35.9–48.0)	<0.0001	405	71.8 (66.6–81.4)	<0.0001
**Combinations of process changes and reduction in activity durations**									
P1, P2, P3	2,984	43.7 (42.2–46.3)	<0.0001	856	36.6 (34.9–44.6)	<0.0001	405	61.3 (56.3–72.6)	<0.0001
R1, R2	3,022	45.7 (42.0–49.1)	<0.0001	780	36.7 (35.4–48.0)	<0.0001	405	65.3 (62.8–70.6)	<0.0001
P1, P2, P3, R1, R2	2,984	40.6 (39.1–43.2)	<0.0001	856	33.6 (32.4–44.6)	<0.0001	405	52.9 (49.8–61.2)	<0.0001

*DNT, door-to-needle time; EMS, emergency medical services; tPA, tissue plasminogen activator; n, total number of cases; P1, patients arriving via EMS remain on EMS stretcher to imaging; P2, administration of tPA in imaging area; P3, pre-registration of patients arriving via EMS; R1, reduce treatment decision time by 35%; R2, reduce tPA administration preparation time by 35%. Analysis was performed using the Mann-Whitney U-test comparing DNT data from the changes that were implemented to the respective site's baseline DNT data*.

### Test Scenario Experiment Results

**Figure 2:**
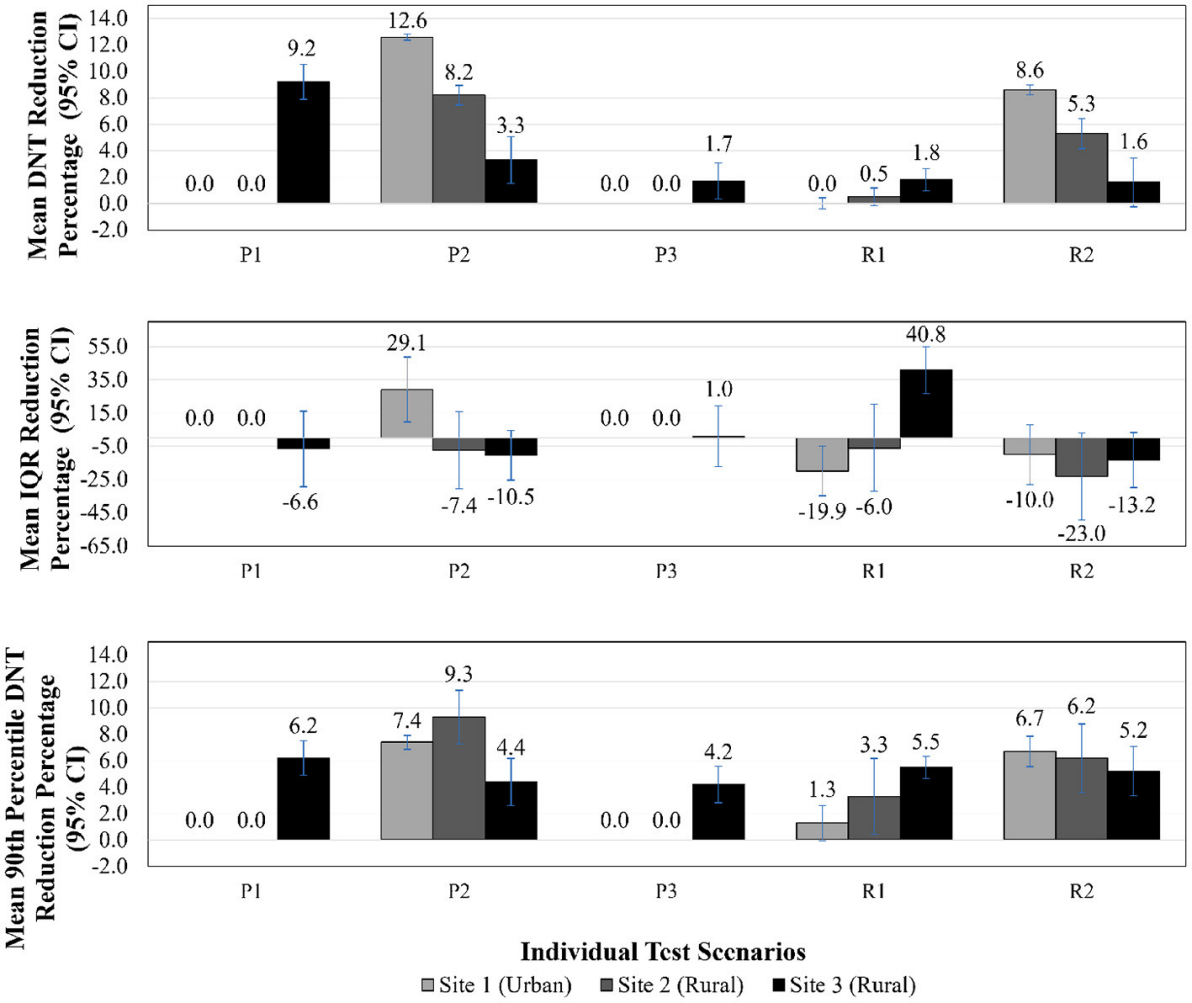
DNT reduction, IQR reduction, 90^th^ percentile DNT reduction—individual test scenario results. DNT, door-to-needle time; CI, confidence interval; IQR, interquartile range; P1, patients arriving via EMS remain on EMS stretcher to imaging; P2, administration of tPA in imaging area; P3, pre-registration of patients arriving via EMS; R1, reduce treatment decision time by 35%; R2, reduce tPA administration preparation time by 35%.

**Figure 3:**
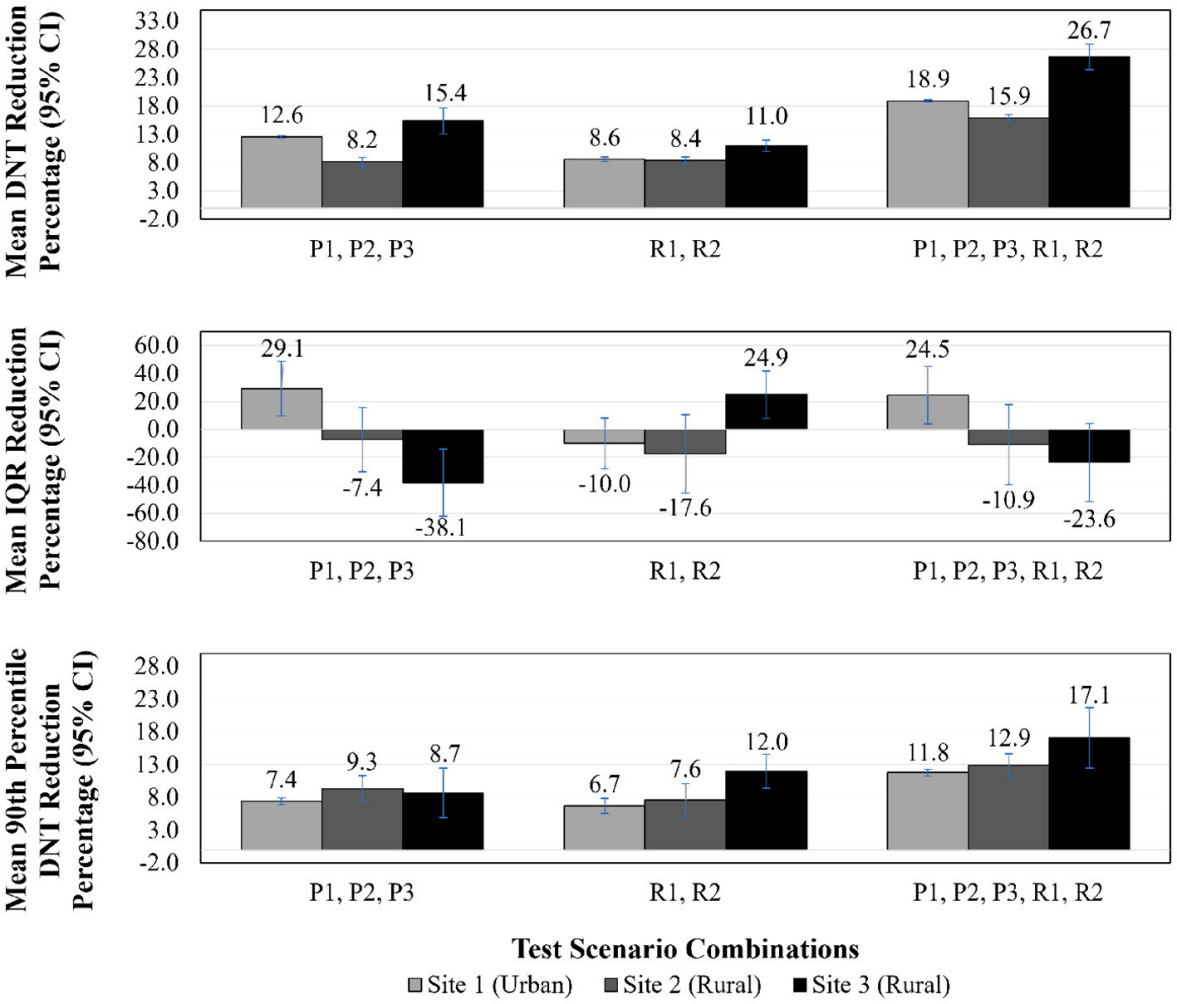
DNT reduction, IQR reduction, 90^th^ percentile DNT reduction—test scenario combinations results. DNT, door-to-needle time; CI, confidence interval; IQR, interquartile range; P1, patients arriving via EMS remain on EMS stretcher to imaging; P2, administration of tPA in imaging area; P3, pre-registration of patients arriving via EMS; R1, reduce treatment decision time by 35%; R2, reduce tPA administration preparation time by 35%.

The results of all scenario experiments are described below and summarized in Tables 3, 4 and Figures 2, 3. Table 3 includes median DNT and IQR range results for each scenario experiment, and the total number of cases used in the calculations for median DNT for each scenario.

**Table 4:** 90^th^ percentile DNT results by scenario for Site 1, Site 2, and Site 3.

	**Site 1 (Urban)**		**Site 2 (Rural)**		**Site 3 (Rural)**	
	***n***	**90^**th**^ Percentile DNT (min)**	***n***	**90^**th**^ Percentile DNT (min)**	***n***	**90^**th**^ Percentile DNT (min)**
**Process baseline**	3,021	71.3	780	62.8	405	92.9
**Process changes**						
P1	3,021	Current baseline	780	Current baseline	405	91.3
P2	2,984	66.1	856	58.9	405	92.0
P3	3,021	Current baseline	780	Current baseline	405	92.8
**Reduction in activity durations**						
R1	3,021	71.3	780	62.4	405	89.2
R2	3,022	67.3	780	60.5	405	91.5
**Combinations of process changes and reduction in activity durations**						
P1, P2, P3	2,984	66.1	856	58.9	405	91.1
R1, R2	3,022	67.3	780	59.3	405	84.8
P1, P2, P3, R1, R2	2,984	63.0	856	56.2	405	82.7

*DNT, door-to-needle time; EMS, emergency medical services; tPA, tissue plasminogen activator; n, total number of cases; P1, patients arriving via EMS remain on EMS stretcher to imaging; P2, administration of tPA in imaging area; P3, pre-registration of patients arriving via EMS; R1, reduce treatment decision time by 35%; R2, reduce tPA administration preparation time by 35%*.

Figures 2, 3 illustrate DNT reduction percentage, IQR reduction percentage, and 90th percentile DNT reduction percentage results for all three sites for all individual test scenarios, and combinations of scenarios, respectively. The 90^th^ percentile DNT results are summarized in Table 4. Note that process changes (P1, P2, or P3) tested that resulted in DNT reductions of zero signify the site was already implementing that scenario as part of their baseline.

*Patients Arriving via EMS Remain on EMS Stretcher to Imaging (P1)*—As Site 1 and Site 2 were currently implementing this process as their baseline, those sites did not experience an improvement in DNT. This process change did benefit Site 3, and led to the largest improvement for Site 3 across all individual test scenarios. The DNT at Site 3 was reduced to 66.4 (IQR 63.6–75.7) min from a median of 74.0 (IQR 70.8–82.6) min, translating to a 9.2% (95% CI 7.9–10.5%) improvement in DNT compared to the respective site baseline.

*Administration of tPA in Imaging Area (Regular and Out of Hours) (P2)*—Administration of tPA to patients in the imaging area resulted in a reduction of the median DNT for Site 1, Site 2, and Site 3 to 43.7 (IQR 42.2–46.3) min, 36.6 (IQR 34.9–44.6) min, and 71.0 (IQR 66.0–80.3) min, respectively. This scenario was the most impactful individual test result for Site 1 and Site 2, leading to a reduction of 12.6% (95% CI 12.4–12.8%) and 8.2% (95% CI 7.5–9.0%), respectively. It was anticipated that Site 1 would show the largest improvement for this scenario, as Site 1 has the longest travel time to the ED from imaging since it is large tertiary care center.

*Pre-registration of Patients Arriving *via* EMS (P3)*—As Site 1 and Site 2 include this process in their baseline, those sites did not experience an improvement in DNT. This process change resulted in a DNT of 71.9 (IQR 68.6–80.9) min and a reduction of 1.7% (95% CI 0.3–3.1%) at Site 3.

*Reduce Treatment Decision Time by 35% (R1)*—Physician comfort with treatment with tPA results in a shorter amount of time for making the treatment decision. The reduction in decision time did not reduce Site 1's DNT, highlighting that a bottleneck was present elsewhere. Scenario R1 also had little impact on Site 2, with a 0.5% (95% CI −0.1–1.2%) improvement in DNT. Site 3 experienced a 1.8% (95% CI 1.0–2.7%) improvement, with a DNT of 72.3 (IQR 70.0–76.0) min.

*Reduce tPA Administration Preparation Time by 35% (R2)*—This reduction in activity duration scenario benefitted Site 1 and Site 2 to a greater degree than did R1. The DNT was reduced for Site 1 by 8.6% (95% CI 8.2–9.0%) with a DNT of 45.7 (IQR 42.0–49.1) min. Site 2 improved their DNT by 5.3% (95% CI 4.2–6.5%) with an output of 37.8 (IQR 35.9–48.0) min. The impact of R2 was comparable to R1 for Site 3, with DNT reduction of 1.6% (95% CI −0.2–3.5%) and a DNT of 71.8 (IQR 66.6–81.4) min.

*Combination of P1, P2, P3*—When combining all process change scenarios, Site 3 saw a 15.4% (95% CI 13.1–17.7%) reduction with a DNT of 61.3 (IQR 56.3–72.6) min from a baseline median of 74.0 (IQR 70.8–82.6) min. As Site 1 and Site 2 were currently implementing P1 and P3, their respective results are identical to their P2 results as that was the only new process change for those sites.

*Combination of R1, R2*—Combining the two reductions of activity duration scenarios benefited Site 3 far more than Site 1 and Site 2, whose improvements in this case were largely attributable to R2. The combination of R1 and R2 was equally, or more, effective in terms of improving DNT when compared to being individually tested, due to the close relationship of these activities and the bottleneck formed when adjusted individually. The resulting DNT for Site 1, Site 2, and Site 3 were 45.7 (IQR 42.0– 49.1) min, 36.7 (IQR 35.4–48.0) min, and 65.3 (IQR 62.8–70.6) min, respectively.

*Combination of P1, P2, P3, R1, R2*—As anticipated, the lowest DNT for each site was achieved by combining all test scenarios, which resulted in the following DNTs: Site 1 was reduced by 18.9% (95% CI 18.6–19.1%) to 40.6 (IQR 39.1–43.2) min, Site 2 by 15.9% (95% CI 15.3–16.4%) to 33.6 (IQR 32.4–44.6) min, and Site 3 by 26.7% (95% CI 24.5–28.9) to 52.9 (IQR 49.8–61.2) min.

In addition to median DNT and DNT reduction percentages results, Figures 2, 3 illustrate IQR reduction percentages and 90^th^ percentile DNT reduction percentages results which further define the spread of the DNT results. As shown in Figures 2, 3, Site 2 did not experience a reduction in IQR with individual or combinations of test scenarios. It can be seen that the following individual test scenarios led to the largest reduction in IQR for Site 1 and Site 3, respectively: P2 with a reduction in IQR of 29.1% (95% CI 9.7–48.5%), and R1 with a reduction in IQR of 40.8% (95% CI 26.7–54.8%). Site 1 experienced a reduction in IQR in all test scenarios combinations involving P2, such as: P1, P2, P3 (reduction of 29.1%, 95% CI 9.7–48.5%), and P1, P2, P3, R1, R2 (IQR reduction of 24.5%, 95% CI 4.0–45.0%). Site 3 also had a reduced IQR for test scenario combination R1, R2 (IQR reduction of 24.9%, 95% CI 7.9–42.0%). The majority of individual and combinations of test scenarios resulted in an increased IQR. The lowest 90^th^ percentile DNT experienced in individual test scenarios for Site 1, Site 2 and Site 3, respectively, as shown in Table 4, are the following: 66.1 min resulting from P2 (reduction of 7.4%, 95% CI 6.9–8.0%), 58.9 min resulting from P2 (reduction of 9.3%, 95% CI 7.3–11.3%), and 89.2 min resulting from R1 (reduction of 5.5%, 95% CI 3.0–7.9%). The lowest 90^th^ percentile DNT results when considering all individual and combinations of test scenarios were achieved by combining all three process changes and two reduction in activity durations for all sites. When combining all test scenarios, the following 90^th^ percentile DNT improvements were experienced: Site 1 was reduced from 71.3 to 63.0 min (reduction of 11.8%, 95% CI 11.3–12.3%), Site 2 was reduced from 62.8 to 56.2 min (reduction of 12.9%, 95% CI 11.2–14.7%), and Site 3 was reduced from 92.9 to 82.7 min (reduction of 17.1%, 95% CI 12.5–21.7%).

## Discussion

The developed conceptual framework expands upon published works (33) to include details extending past key treatment activities, and highlights when delays may be encountered. The framework can be used to promote discussion among healthcare professionals treating AIS patients by facilitating brainstorming methods to streamline processes. It is important when creating a framework to represent urban and rural settings, to accommodate for treatment process differences between them. Low volumes of thrombolysis treatment in rural hospitals makes it challenging to trial process changes, making DES a beneficial tool to incorporate process improvement. A recent study that reduced DNTs across an entire population further illustrated the challenge in improving DNT in rural settings as rural hospitals were only able to reduce their DNTs to a median of 54 min from 84 min after the year-long study (11). Discrete-event simulation modeling can provide hospitals with an initial assessment of treatment changes to then implement those that will have the largest impact, without needing to commit resources for trial. Using a DES model allows the potential impact of process improvements to be quantified, providing an initial assessment pre-implementation, and evidence for further trial. The included case studies provided context for the results, demonstrating the impact of treatment changes in a group of hospitals with varying characteristics which allows the findings from the developed model to be applicable elsewhere with no additional work required. Although, the model could be modified for further assessment of treatment changes at additional sites.

The study shows that the urban and rural sites can benefit from process improvements, by implementing process changes or reducing activity durations. As anticipated, the applied test scenarios resulted in a larger DNT reduction in Site 3, as it was not implementing any of the scenarios and had the longest median DNT. It should be noted that Site 3's DNT is capable of being lower than this study has illustrated, if the reduction in activity duration scenarios reduced durations to a set value, vs. reducing the site's current durations by 35%. The individual and combination testing of the reduction in activity duration scenarios illustrated that reducing one activity duration can create a bottleneck elsewhere, and in situations with parallel activities, all activities on the critical path must be reduced to see an impact. In addition to focusing on the impact of treatment changes on median DNT, it is important to also acknowledge the 90^th^ percentile DNT (43). The 90^th^ percentile DNT metric defines the timeframe in which the majority of patients are treated, and according to the current Canadian Guidelines the 90^th^ percentile DNT should be 60 min (44). The study results show that Site 2 can achieve this standard with minimal process improvements, while Site 1 is close with the implementation of all test scenarios.

The DNT improvements from the test scenarios using the DES model resulted in less reduction than reported in the literature, where reductions of 20–40% have resulted from implementing similar process changes (11, 12, 35, 37, 38, 40). There are many reasons for this, as the implementation of a process change may result in activity duration reductions across the entire treatment process. The magnitude of DNT improvements shown in the literature for implemented changes, not simulated, may be attributable to a behavioral component of implementation, meaning greater reductions in DNT may be realized through implementation. Finding solutions using DES modeling that result in reduced DNTs has the potential to improve clinical outcomes due to the established relationship between treatment times and patient outcomes (8, 9). Additional hospital-based strategies such as: single-call activation of care team, rapid acquisition of brain imaging, point of care International Normalized Ratio (INR) testing, rapid access to tPA, and not requiring written informed consent (14, 35, 40), should be incorporated for further improvements.

The benefit of trialing process changes within a DES model is clear, although there are challenges involved in developing an appropriately accurate model to represent the system. A study focusing on the development of hyperacute stroke care frameworks summarized the main two difficulties concisely: (1) modelers must understand clinical concepts and have the ability to grasp the pathway logistics; and (2) translate that knowledge into a model for use of testing (33). This highlights the importance of hyperacute stroke frameworks and the need for providing as much detail as possible for logistics to be understood thoroughly. Only including key activities in these frameworks and models may limit the extent of the improvement. Additionally, access to the required data, including activity durations, to establish a reasonable representation of the system is a challenge. To have an accurate description of activity durations, data collection studies, such as time studies, would be beneficial. Time studies are useful in providing required data in areas such as: activity durations based on triage category (45), and clinician work time allocation and interruptions (46), enabling further model accuracy. It is advantageous for healthcare professionals treating AIS patients to be aware of this data, as awareness of benchmarks could lead to DNT improvements with a team culture to provide rapid care and establish a continuous improvement mindset.

### Limitations

A limitation of this study is that the conceptual framework was based on only three hospitals with a small sample size of participants involved at each site in a single Canadian province (13). Furthermore, only two rural sites were studied, meaning the conceptual framework presented may not address all urban-rural treatment differences. It would be advisable to incorporate additional urban and rural hospitals to ensure variations in treatment are encompassed fully. As the conceptual framework was based on interviews conducted in a single Canadian province, the framework likely includes inefficiencies in their system. The process can have additional efficiencies by implementing the following: completing the neurological assessment while the patient is traveling to imaging, having the tPA mixed by the time the non-contrast CT has occurred, and administrating tPA after the non-contrast CT but before the CTA. It is important to note that the model assumes 100% of stroke protocol compliance and implementation of test scenarios. For example, when testing the process change of taking a patient to imaging on the EMS stretcher, personnel dependent variation in compliance or variation based on patient factors may dictate the need to move the patient to an ED bay for stabilization prior to imaging was not incorporated. The main limitation of the DES model was the lack of available data detailing activity durations. The model would benefit by employing a time study and replacing estimated durations with collected data for improved accuracy. A second limitation is the use of assumptions and simplifications. The scope of the study was limited by focusing on thrombolysis and not including EVT. It is recognized that rural hospitals face additional challenges, such as arranging transfer of patients for EVT, deserving further study.

## Conclusion

The conceptual framework developed in this study defines treatment pathways for AIS patients receiving thrombolysis treatment in urban or rural hospitals. The detail of the framework aims to reduce the gap between clinical knowledge and modeler perceptions regarding the logistics of the intra-hospital aspect of the thrombolysis process. Several process improvements were shown to have a positive impact on median DNT and 90^th^ percentile DNT in urban and rural settings. Our modeling shows that of the three included process changes, the most significant median DNT improvement at rural hospitals resulted from ensuring patients arriving via EMS remain on the EMS stretcher to imaging, while urban sites benefit greater from administering tPA in the imaging area. Reducing the durations of activities found on the critical path will result in further DNT improvements. The lowest median DNTs and 90^th^ percentile DNTs results were achieved by combining all three process changes and two reduction in activity durations for all sites. Significant DNT improvements are achievable in urban and rural settings by combining process changes and reducing activity durations. Based on the results of this study, similar sites can feel confident in achieving desired outcomes when implementing these process improvements. Discrete-event simulation modeling of acute stroke processes can be expanded to develop more accurate models to reveal the ideal processes to achieve the most efficient treatment for patients.

## Data Availability Statement

The original contributions presented in the study are included in the article/supplementary material, further inquiries can be directed to the corresponding author/s.

## Author Contributions

TB: study design, DES modeling, data analysis, preparation of figures and tables, preparation of the first and revised drafts of the manuscript, as well as final editing, and formatting. DV: revision of manuscript for intellectual content. JB: input into the following areas: data analysis, model verification, sensitivity analysis, statistical analysis, as well as revision of manuscript for intellectual content. NK: study design, input into data analysis, editing, formatting, and revision of manuscript for intellectual content. All authors contributed to the article and approved the submitted version.

## Funding

This work was funded by Canadian Institutes of Health Research (CIHR) Project Grant (PJT-169124) held by NK.

## Conflict of Interest

NK: Founder and part equity owner of DESTINE Health. Principal Investigator for CIHR grant that funded this work. The remaining authors declare that the research was conducted in the absence of any commercial or financial relationships that could be construed as a potential conflict of interest.

## Publisher's Note

All claims expressed in this article are solely those of the authors and do not necessarily represent those of their affiliated organizations, or those of the publisher, the editors and the reviewers. Any product that may be evaluated in this article, or claim that may be made by its manufacturer, is not guaranteed or endorsed by the publisher.
