# Supplementary material for: Discrete-Event Simulation to Model the Thrombolysis Process for Acute Ischemic Stroke Patients at Urban and Rural Hospitals
Source: Front Neurol. 2021 Oct 29;12:746404. doi: 10.3389/fneur.2021.746404 (PMC8586711; doi:10.3389/fneur.2021.746404)
Supplement: Supplementary file 1 [file Data_Sheet_1.PDF]

Model Details

Table S1: Model Baseline and Test Scenario Activity Durations for Each Site. ED: Emergency Department, IV: Intravenous, CT: Computed Tomography, CTA: Computed Tomography Angiography, tPA: Tissue Plasminogen Activator, EMS: Emergency Medical Services, PV: Private Vehicle, TRIA: Triangular Distribution, UNIF: Uniform Distribution, DISC: Discrete Distribution, P1: Patients Arriving via EMS Remain on EMS Stretcher to Imaging, P2: Administration of tPA in Imaging Area, P3: Pre-Register Patients Arriving via EMS, R1: Reduce Treatment Decision Time by 35%, R2: Reduce tPA Administration Preparation Time by 35%. EMS indicates the patient arrived to the hospital via EMS with the stroke protocol activated pre-arrival, PV indicates the patient arrived via private vehicle.

**Activity**	**Site 1 (Urban)** (min)	**Site 2 (Rural)** (min)	**Site 3 (Rural)** (min)	**Data Source** (all sites)
Wait to be Triaged	EMS: N/A	EMS: N/A	EMS: N/A	Interviews with Healthcare Professionals (13)
	PV: TRIA(5, 10, 15)	PV: TRIA(5, 10, 15)	PV: TRIA(5, 10, 15)	
Suspected Stroke Identified by Triage Nurse	EMS: N/A	EMS: N/A	EMS: N/A	
	PV: TRIA(3, 4, 5)	PV: TRIA(3, 4, 5)	PV: TRIA(3, 4, 5)	
Physician Flagged Immediately	EMS: N/A	EMS: N/A	EMS: N/A	
	PV: TRIA(1, 2, 3)	PV: TRIA(1, 2, 3)	PV: TRIA(1, 2, 3)	
Physician Assesses Patient to Determine Stroke Protocol Activation	EMS: N/A	EMS: N/A	EMS: N/A	
	PV: TRIA(2, 4, 5)	PV: TRIA(2, 3, 5)	PV: TRIA(3, 4, 5)	
Collect Bloodwork, Vitals, and IVs	TRIA(5, 7, 10.5)	Bloodwork: TRIA(2, 3, 4) Vitals: TRIA(3, 4, 5)	Bloodwork: TRIA(2, 3, 4) Vitals and IVs: TRIA(5, 6, 7)	
Neurological Assessment	TRIA(3, 5, 10)	TRIA(3, 5, 6)	TRIA(5, 7, 9)	
Registration	EMS: UNIF(0.5, 1)	EMS: UNIF(0.25, 0.5)	EMS: UNIF(2, 3)<br><br>*Scenario P3* – UNIF(0.25, 0.50)	
	PV: UNIF(2, 3)	PV: UNIF(2, 3)	PV: UNIF(2, 3)	

**Activity**	**Site 1 (Urban)** (min)	**Site 2 (Rural)** (min)	**Site 3 (Rural)** (min)	**Data Source** (all sites)
Transfer Patient to ED Bay	EMS: N/A	EMS: N/A	EMS: UNIF(6, 9)<br>*Scenario P1* – 0	Interviews with Healthcare Professionals (13)
	PV: UNIF(3, 5)	PV: UNIF(3, 5)	PV: UNIF(3, 5)	
Travel to Imaging	UNIF(3, 5)	UNIF(1, 2)	UNIF(0.5, 1)	
Transfer Patient to Scanner	UNIF(2, 4)	UNIF(2, 4)	UNIF(2, 4)	
CT Technologist Travelling to Hospital (Out of Hours Only)	N/A	N/A	EMS: TRIA(0, 5, 10)	
			PV: TRIA(10, 15, 20)	
CT Technologist Gets Scanner Ready (Out of Hours Only)	N/A	N/A	10	
CT Technologist Plans Images	UNIF(1, 2)	UNIF(1, 2)	UNIF(2, 3)	
CT	1	1	1	
CTA	5	5	5	
Additional Scan	1.5	2	N/A	
Transfer Patient to Stretcher	UNIF(2, 4)	UNIF(2, 4)	UNIF(2, 4)	
Review Patient History	EMS: UNIF(0, 2)	EMS: UNIF(0, 2)	EMS: DISC(0.5, UNIF(0, 5), 1.0, UNIF(5, 8))	
	PV: UNIF(5, 8)	PV: UNIF(5, 8)	PV: UNIF(5, 8)	
Neurologist Travelling to Hospital (Out of Hours Only)	EMS: TRIA(0, 5, 10)	N/A	N/A	
	PV: TRIA(10, 15, 20)	N/A	N/A	
Receive CT Report	TRIA(3, 4, 5)	TRIA(3, 4, 5)	TRIA(5, 8, 10)	

**Activity**	**Site 1 (Urban)** (min)	**Site 2 (Rural)** (min)	**Site 3 (Rural)** (min)	**Data Source** (all sites)
Make Treatment Decision	TRIA(5, 6, 9)<br><br>*Scenario R1* – 0.65 * TRIA(5, 6, 9)	TRIA(5, 8, 20)<br><br>*Scenario R1* – 0.65 * TRIA(5, 8, 20)	TRIA(10, 20, 40)<br><br>*Scenario R1* – 0.65 * TRIA(10, 20, 40)	Interviews with Healthcare Professionals (13)
Wait for Lab Results	DISC(0.9, 0, 1.0, TRIA(10, 15, 40))	DISC(0.9, 0, 1.0, TRIA(10, 15, 30))	DISC(0.9, 0, 1.0, (TRIA(25,30,40) * (Regular Hours) + (TRIA(60,75,90) * (Out of Hours)	
Travel to Treatment Location	0 * (Regular Hours) + UNIF(3, 5) * (Out of Hours)<br><br>*Scenario P2* – 0	UNIF(1, 2)<br><br>*Scenario P2* – 0	UNIF(0.5, 1)<br><br>*Scenario P2* – 0	
tPA Administration Preparation	UNIF(8, 10) * (Regular Hours) + UNIF(10, 15) * (Out of Hours)<br><br>*Scenario P2* – UNIF(8, 10)<br><br>*Scenario R2* – 0.65 * [UNIF(8, 10) * (Regular Hours) + UNIF(10, 15) * (Out of Hours)]	UNIF(8, 10)<br><br>*Scenario P2* – UNIF(5, 8)<br><br>*Scenario R2* – 0.65 * UNIF(8, 10)	Bed Huddle: TRIA(3, 5, 7)<br><br>*Scenario P2* – TRIA(3, 4, 5)<br><br>*Scenario R2* – 0.65 * TRIA(3, 5, 7) UNIF(15, 20)<br><br>tPA Administration Preparation: *Scenario P2* – UNIF(8, 15)<br><br>*Scenario R2* – 0.65 * UNIF(15, 20)	
Obtain Patient Consent	TRIA(3, 5, 7)	TRIA(3, 5, 7)	TRIA(5, 8, 12)	
Mix tPA	UNIF(1, 2)<br><br>*Scenario R2* – 0.65 * UNIF(1, 2)	UNIF(2, 3)<br><br>*Scenario R2* – 0.65 * UNIF(1, 2)	UNIF(1, 4)<br><br>*Scenario R2* – 0.65 * UNIF(1, 2)	
Administer tPA Bolus	UNIF(1, 2)	UNIF(1, 2)	UNIF(1, 2)	

Table S2: Model Arrival Schedule for Each Site.

Time Period	Site 1 (Urban) Arrival Rate	Site 2 (Rural) Arrival Rate	Site 3 (Rural) Arrival Rate
12:00am – 2:00am	0.00428	0.00116	0.00058
2:00am – 4:00am	0.00321	0.00087	0.00043
4:00am – 6:00am	0.00321	0.00087	0.00043
6:00am – 8:00am	0.00642	0.00173	0.00087
8:00am – 10:00am	0.01391	0.00376	0.00188
10:00am - 12:00pm	0.01819	0.00491	0.00246
12:00pm – 2:00pm	0.01926	0.00520	0.00260
2:00pm – 4:00pm	0.01712	0.00462	0.00231
4:00pm – 6:00pm	0.01712	0.00462	0.00231
6:00pm – 8:00pm	0.01605	0.00433	0.00217
8:00pm – 10:00pm	0.01070	0.00289	0.00144
10:00pm – 12:00am	0.00856	0.00231	0.00116
	**Data Source**		
	Arrival rates calculated using provided Site 1 patient arrival data.	Site 1 arrival rates adjusted using aggregate data to consider the difference in ischemic patient volumes encountered between Site 1 and Site 2.	Site 1 arrival rates adjusted using aggregate data to consider the difference in ischemic patient volumes encountered between Site 1 and Site 3.

Model Verification

The simulation model was verified using the several techniques detailed below, which was completed using data from Site 1.

The following techniques were utilized to complete model verification:

- *Analysis of code:* Model component code was carefully reviewed to ensure accuracy of the intended outcome.

- *Output analysis:* Patient attributes and DNT were recorded into Excel for data analysis, verifying that model output was appropriate given the patient's details.

- *Comparison of input to model output:* The Chi-Square goodness-of-fit test was used to determine statistical significance of the desired arrival schedule to the model output. The results are detailed in Table 2 in the manuscript, along with the pathway type and time period breakdowns. Activity durations were verified based on pathway type and time period distinctions using data recorded in Excel, comparing desired distributions to model output, summarized in Table S3.

Table S3: Site 1 Model Verification of Activity Durations. ED: Emergency Department, IV: Intravenous, CT: Computed Tomography, CTA: Computed Tomography Angiography, tPA: Tissue Plasminogen Activator, EMS: Emergency Medical Services, PV: Private Vehicle, TRIA: Triangular Distribution, UNIF: Uniform Distribution, DISC: Discrete Distribution. EMS indicates the patient arrived to the hospital via EMS with the stroke protocol activated pre-arrival, PV indicates the patient arrived via private vehicle.

**Activity**	**Desired Distribution** (min)<br>• Constant<br>• UNIF(Minimum, Maximum)<br>• TRIA(Minimum, Most Likely, Maximum)	**Model Output** (min)<br>• Constant<br>• (Minimum, Maximum)<br>• (Minimum, Average, Maximum)
Wait to be Triaged	EMS: N/A	EMS: 0
	PV: TRIA(5, 10, 15)	PV: (5.2, 10.0, 14.9)
Suspected Stroke Identified by Triage Nurse	EMS: N/A	EMS: 0
	PV: TRIA(3, 4, 5)	PV: (3.0, 4.0, 5.0)
Physician Flagged Immediately	EMS: N/A	EMS: 0
	PV: TRIA(1, 2, 3)	PV: (1.0, 2.0, 3.0)
Physician Assesses Patient to Determine Stroke Protocol Activation	EMS: N/A	EMS: 0
	PV: TRIA(2, 4, 5)	PV: (2.0, 3.7, 5.0)

Activity	**Desired Distribution** (min)<br>• Constant<br>• UNIF(Minimum, Maximum)<br>• TRIA(Minimum, Most Likely, Maximum)	**Model Output** (min)<br>• Constant<br>• (Minimum, Maximum)<br>• (Minimum, Average, Maximum)
Collect Bloodwork, Vitals, and IVs	TRIA(5, 7, 10.5)	(5.1, 7.5, 10.4)
Neurological Assessment	TRIA(3, 5, 10)	(3.1, 6.0, 9.9)
Registration	EMS: UNIF(0.5, 1)	EMS: (0.5, 1.0)
	PV: UNIF(2, 3)	PV: (2.0, 3.0)
Transfer Patient to ED Bay	EMS: 0.0	EMS: 0.0
	PV: UNIF(3, 5)	PV: (3.0, 5.0)
Travel to Imaging	UNIF(3, 5)	(3.0, 5.0)
Transfer Patient to Scanner	UNIF(2, 4)	(2.0, 4.0)
CT Technologist Plans Images	UNIF(1,2)	(1.0, 2.0)
CT	1	1.0
CTA	5	5.0
CT Perfusion	1.5	1.5
Transfer Patient to Stretcher	UNIF(2, 4)	(2.0, 4.0)
Review Patient History	EMS: UNIF(0, 2)	EMS: (0.0, 2.0)
	PV: UNIF(5, 8)	PV: (5.0, 8.0)
Neurologist Travelling to Hospital (Out of Hours Only)	EMS: TRIA(0, 5, 10)	EMS: (0.1, 5.0, 9.8)
	PV: TRIA(10, 15, 20)	PV: (10.1, 14.8, 19.5)
Receive CT Report	TRIA(3, 4, 5)	(3.0, 4.0, 5.0)
Make Treatment Decision	TRIA(5, 6, 9)	(5.1, 6.7, 9.0)
Wait for Lab Results	DISC(0.9, 0, 1.0, TRIA(10, 15, 40))	0, (10.7, 22.0, 37.9)
Travel to Treatment Location	0 * (Regular Hours) + UNIF(3, 5) * (Out of Hours)	Regular Hours: 0.0<br>Out of Hours: (3.0, 5.0)
tPA Administration Preparation	UNIF(8, 10) * (Regular Hours) + UNIF(10, 15) * (Out of Hours)	Regular Hours: (8.0, 10.0)<br>Out of Hours: (10.0, 15.0)

Activity	**Desired Distribution** (min)<br>• Constant<br>• UNIF(Minimum, Maximum)<br>• TRIA(Minimum, Most Likely, Maximum)	**Model Output** (min)<br>• Constant<br>• (Minimum, Maximum)<br>• (Minimum, Average, Maximum)
Obtain Patient Consent	TRIA(3, 5, 7)	(3.0, 5.0, 6.9)
Mix tPA	UNIF(1, 2)	(1.0, 2.0)
Administer tPA Bolus	UNIF(1, 2)	(1.0, 2.0)

The following techniques were utilized to complete extended model verification:
• *Use of site-specific models and real site data:* The developed DES was applied to the three included sites for further verification purposes. The model was applied using each site's process maps from the qualitative study (13), using real site median DTN data from Nova Scotia Health for comparison.

• *Animation:* The model was run using animation and display of patient attributes to allow the modeller to observe the entity travelling through the system to ensure appropriate behaviour.

• *Spreadsheet calculations:* Process calculations were completed in Excel using the activity distribution expected value equations, pathway type breakdown, and regular and out of hour percentages defined in Table S4, with the calculation results compared to the model output summarized in Table S5.

Table S4: Definitions used for Site 1 Extended Model Verification Calculations. EMS: Emergency Medical Services, PV: Private Vehicle.

**Activity Duration Expected Value Calculations**	
Distribution	*Expected Value*
Constant (a)	a
Uniform<br>(Minimum a, Maximum b)	$\frac{(a+b)}{2}$
Triangular<br>(Minimum a, Most Likely b, Maximum c)	$\frac{(a+b+c)}{3}$
Discrete<br>(p(a), a, p(a + b), b)	$a * p(a) + b * p(b)$
**Pathway Type Breakdown**	
Pathway Type	80.0 % EMS
	20.0 % PV
**Breakdown of Time Period**	
Definition of Time Period	*Percentage of Arrivals*
Regular Hours	35.4 %
Out of Hours	64.6 %

Table S5: Site 1 Extended Model Verification Results Calculated Using Table S1 Desired Activity Distributions and Table S4 Definitions. DNT: Door-to-Needle Time, CI: Confidence Interval, EMS: Emergency Medical Services, PV: Private Vehicle.

**Pathways**	**Pathway Definition**	**Calculated DNT** (min)	**Overall Calculated DNT** (min)	**Model Output Mean DNT (95 % CI)** (min)
Pathway 1	EMS Regular Hours	42.8	52.3	52.6<br>(52.3 – 53.0)
Pathway 2	EMS Out of Hours	50.3		
Pathway 3	PV Regular Hours	65.7		
Pathway 4	PV Out of Hours	73.2		
**Actual Median DNT** (min)			**Model Output Median DNT (IQR)** (min)	
50.0			50.0 (45.4 – 53.8)	

Sensitivity Analysis

As the test scenarios involved three process changes, it was important to determine how these changes interacted with each other regarding their influence on the outcome measure, median DNT, and whether it was appropriate to test these changes individually. A 2k factorial experiment was completed using Site 3 data and three factors with two settings (low and high) to study the model interactions, shown in Table S6. The model runs for the sensitivity analysis were comprised of 30 replications, with a replication length of one year. The sensitivity analysis results illustrate that the individual factors are considered statistically significant, while the two-way interactions among the factors are not significant, thus it is appropriate to test process changes individually.

Table S6: Site 3 Sensitivity Analysis - 2k Factorial Experiment Minitab Results. EMS: Emergency Medical Services, ED: Emergency Department. P-Value of less than 0.05 is considered statistically significant.

**Number of Levels**	2 (Low and High)	
**Number of Factors**	3 (A, B, and C)	
**Number of Replications**	30	
**Length of Replications**	1 year	
**Factors**	**Settings**	
	Low Setting	*High Setting*
(A) Patients Arriving via EMS Stretcher Type to Travel to Imaging	Patients Arriving via EMS Remain on EMS Stretcher to Imaging	Patients Arriving via EMS Transferred to ED Bay to Travel to Imaging
(B) Treatment Location (Regular & Out of Hours)	Imaging Area	ED
(C) Pre-Registration of Patients Arriving via EMS	Pre-Register Patients Arriving via EMS	Complete Registration of Patients Arriving via EMS Upon Arrival
	**p-Value**	
Linear		
Factor A	0.001	
Factor B	0.002	
Factor C	0.003	
Two-Way Interactions		
Factor A * Factor B	0.446	
Factor A * Factor C	0.221	
Factor B * Factor C	0.351	

Number of Replications

As randomness exists in all simulation results, one simulation replication does not necessarily give the "correct" output. Therefore, it must be determined how many simulation replications are required to achieve a desired confidence interval of the mean DNT. With the goal of outputting results that are sufficiently close to the real DNT values, a number of replications analysis was completed using the following formula, and chosen alpha and half width values shown in Table S7.

$$Number\ of\ Replications\ (n) \geq \left(\frac{s * t_{n-1,\frac{\alpha}{2}}}{\varepsilon}\right)^2$$

A pilot run consisting of 30 replications was completed for each sites' process baseline and a summary of the results is shown in Table S7. The number of replication results shown in Table S7 were calculated based on the chosen alpha and half width values, as well as the calculated sample standard deviation of each sites' outputted DNT values from the pilot run. It is shown that the maximum number of replications required was found to be 10, but for further accuracy the number of replications chosen was determined to be 30.

Table S7: Number of Replications Summarized Results.

	**Site 1 (Urban)**	**Site 2 (Rural)**	**Site 3 (Rural)**
Chosen Alpha (α) (95% Confidence Interval)	0.05	0.05	0.05
Chosen Half Width (ε) (min)	2.00	2.00	2.00
Calculated Sample Standard Deviation (s)	1.37	2.12	2.72
Number of Replications Required (n)	4.75	7.00	9.83
**Number of Replications Required (n) (Rounded to Next Whole Number)**	**5.00**	**7.00**	**10.00**
Resulting t Value	3.18	2.45	2.31
Resulting Half Width (ε)	2.00	1.96	2.00

ARENA Simulation

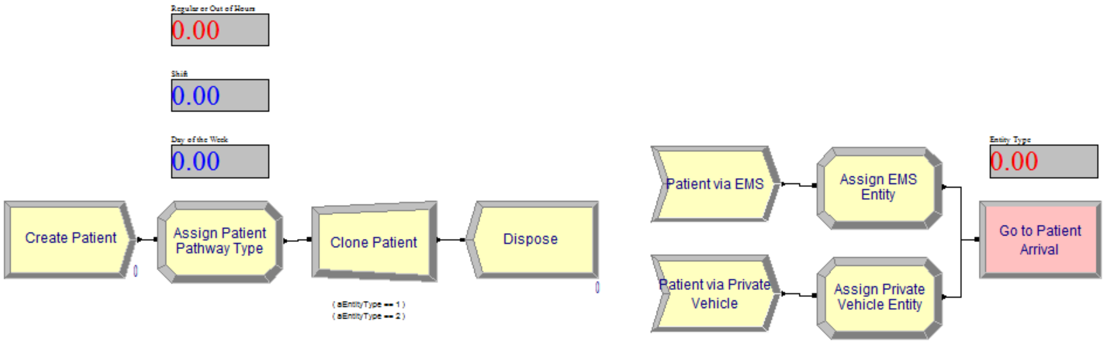

Figure S1: Creation of Patients - ARENA Simulation. EMS: Emergency Medical Services.

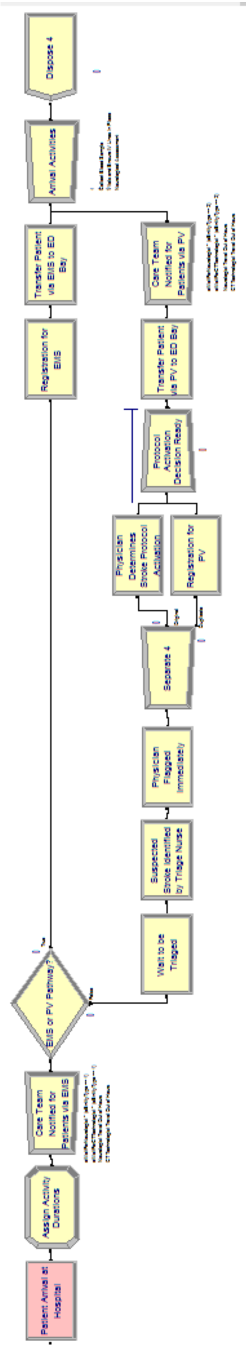

Figure S2: Arrival Activities Part 1 - ARENA Simulation. EMS: Emergency Medical Services, PV: Private Vehicle, ED: Emergency Department.

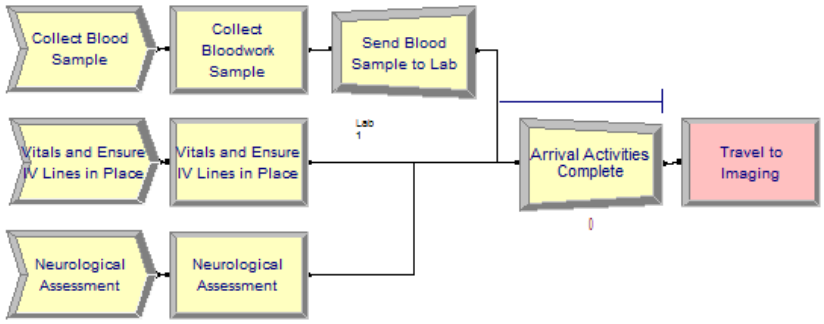

Figure S3: Arrival Activities Part 2 - ARENA Simulation. IV: Intravenous.

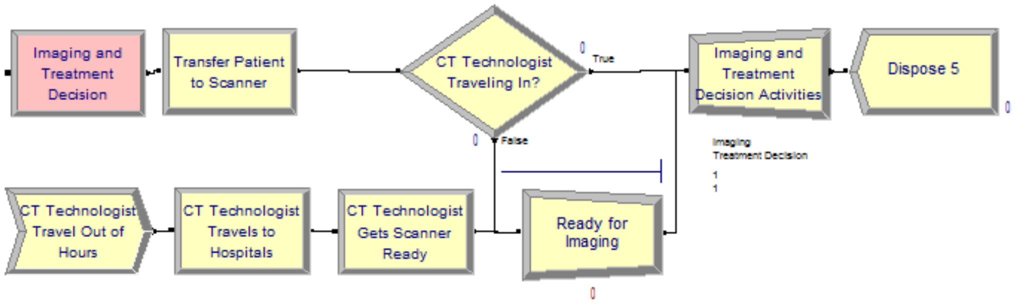

Figure S4: Imaging and Treatment Decision Activities Part 1 - ARENA Simulation. CT: Computed Tomography.

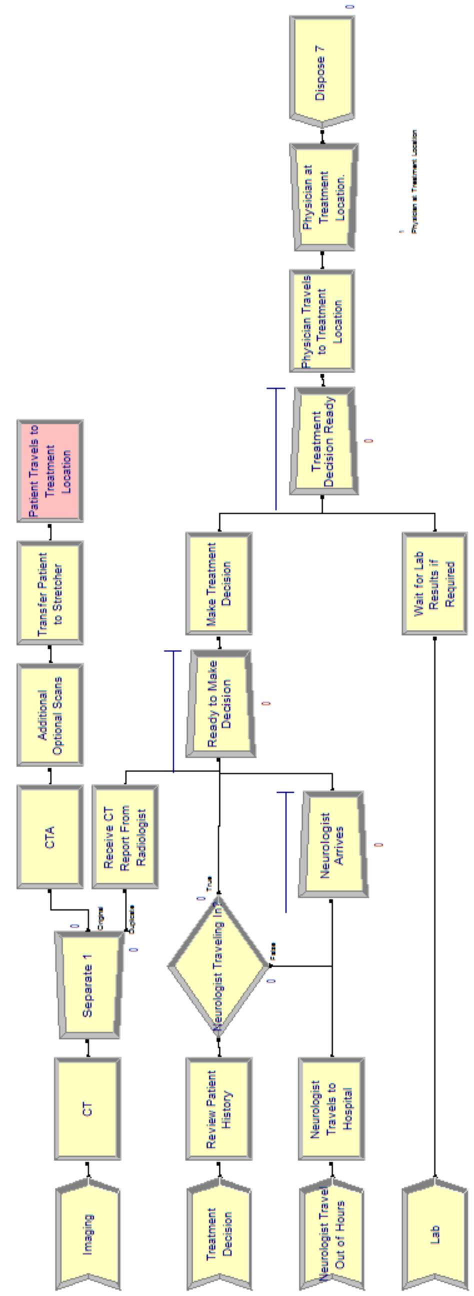

Figure S5: Imaging and Treatment Decision Activities Part 2 - ARENA Simulation. CT: Computed Tomography, CTA: Computed Tomography Angiography.

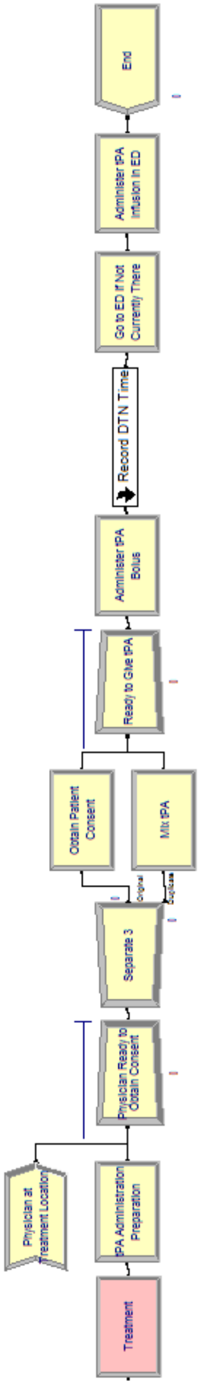

Figure S6: Treatment Activities - ARENA Simulation. tPA: Tissue Plasminogen Activator, DTN: Door-to-Needle, ED: Emergency Department.
